# Emergence of valley selectivity in monolayer tin(ii) sulphide†

**DOI:** 10.1039/c9na00555b

**Published:** 2019-11-11

**Authors:** Eleni Chatzikyriakou, Joseph Kioseoglou

**Affiliations:** Department of Physics, Aristotle University 54124 Thessaloniki Greece elchatz@auth.gr +30 2310 996000

## Abstract

The emergence of valley selectivity in tin(ii) sulphide is explained with the use of density functional theory and the momentum operator matrix elements for the optical transitions. After application of electric stress, the polarization efficiency was found to decrease in the zigzag direction. Wannier functions are further used to derive an effective Tight Binding (TB) model. The velocity matrix elements of the Wannier functions reveal further details about how the p orbitals of Sn and S contribute to optical transitions. Using the TB model in the Wannier basis in a nanoribbon configuration, the bandgap shows an overall decrease as the width of the nanoribbon increases for both zigzag and armchair directions of the structure up to ≈42 Å further presenting opportunities for Optoelectronic applications.

## Abstract 2

Emergence of valley selectivity in tin(ii) sulphide explained with the use of density functional theory and the momentum operator matrix elements for the optical transitions.

## Graphical abstract

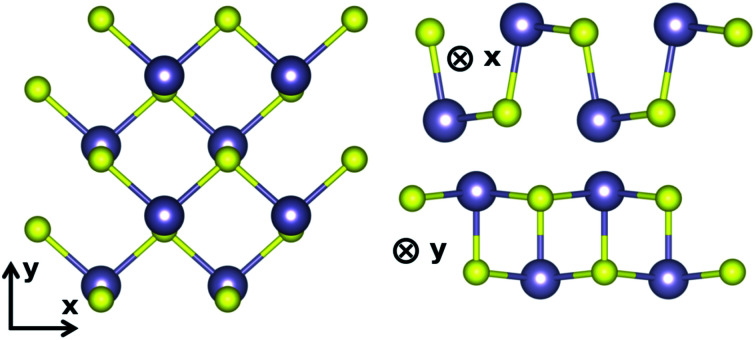

## 1 Introduction

Valleytronics is an attractive alternative to future electronics due to the low power dissipation that they promise.^1^ Furthermore, the possibility of using multiple sources of gating^2^ and transistor modes of operation, including valley qubits,^3^ have also been proposed. Graphene and Transition Metal Dichalcogenides (TMD) MX_2_ (M = Mo, W, X = Se, S) have been extensively studied for the emergence of the relevant physical phenomena tied to the underlying crystal symmetries, including optical selection rules that allow pseudospin up, down and their superposition to be realized.

The interband matrix element, *P*_*n*,*i*_(**k**), of the canonical momentum operator of the Bloch electron undergoing an optical transition, is related to the Berry curvature by,^4,5^1
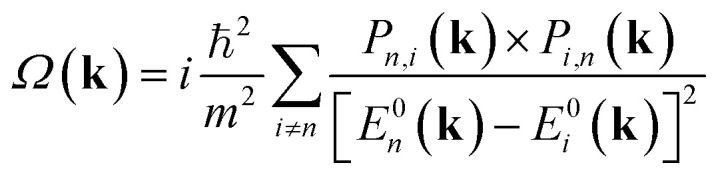
where *i*(*n*) denotes the initial(final) band during the transition, *E*^0^_*i*,*n*_(**k**) is the dispersion of the relevant band. The Berry curvature is a gauge-invariant quantity that reveals geometric properties of the wavefunctions and acts as magnetic field in momentum space. It is peaked at the points of the Brillouin zone where the degree of polarization of the light is maximum. For the case of TMDs, circularly polarized light is required to select each valley,^6^ however, valley selection has further been demonstrated in monochalcogenide systems of type M^III^X^VI^ (M = Ga, In, X = S, Se, Te). In this case, in-plane and out-of-plane polarized light creates bright excitons of even and odd parity respectively.^7^

In this work, we are concerned with valley selection that occurs in a third family of materials, namely M^IV^X^VI^ (M = Ge, Sn, X = S, Se). Due to the valleys that exist in the *x* and *y* directions of their BZ, linearly polarized light in each real space direction induces optical excitations in the equivalent reciprocal space direction.^8–11^ It has also been predicted that, if an electric field is applied in one of these directions, a current with transverse component will be created that results from the absence of mirror symmetry of the valleys that exist in two sets of time-reversed images of one another at the axes of the BZ.^9^ Selection of each valley in this case results from the chosen direction for the application of the electric field and the current appears on the edges of the sample, equivalent to the valley hall effects in TMDs.

We use first principles methods and Wannier functions (WFs), to show how optical selection rules arise from the crystal symmetries, orbital analysis and the matrix elements of the momentum operator. WFs are attractive in this case as they can provide a truncated basis to be used for quantum transport calculations,^12,13^ similar to calculations of valley current using scattering theory.^14^

We focus on monolayer SnS, a material that attracted interest for photovoltaic applications.^15,16^ Furthermore, contrary to some Ga-based monochalcogenides like GaSe and GaTe, photo-induced degradation has not been reported for the equivalent Sn and Ge-based ones,^17^ while it also appears to be more resistant to oxidation states than GeS of the same family.^18^ At the same time, valley-selective linear dichroism has been reported for bulk^10^ and SnS flakes down to 50 nm.^11^

## 2 Density functional theory

Density functional theory calculations were performed in the Quantum Espresso package^19,20^ with both a semi-local (SL) pseudopotential‡‡https://www.quantum-espresso.org/upf_files/Sn.pbe-mt_fhi.UPF, https://www.quantum-espresso.org/upf_files/S.pbe-mt_fhi.UPF. and a projector-augmented wave (PAW) type pseudopotential^21^ with 4d core electrons for Sn in the valence,§§https://www.quantum-espresso.org/upf_files/Sn.pbe-dn-kjpaw_psl.0.2.UPF, https://www.quantum-espresso.org/upf_files/S.pbe-n-kjpaw_psl.0.1.UPF. both of Troullier–Martins type for the interactions between the core and valence electrons. Also, in both cases, Perdew/Burke/Ernzerhof functional was used with the generalized gradient approximation for the exchange correlation potential.^22^

After convergence study, the plane-wave cut-off was set at 65 Ry with 64 bands in both cases. The total energy was considered converged at 0.0001 eV change for the SL pseudopotential and 0.001 eV change for the PAW pseudopotential. The atoms were allowed to relax down to 0.02 eV Å^−1^ maximum force per atom for the SL pseudopotential and 0.001 eV Å^−1^ for the PAW pseudopotential. A Monkhorst–Pack mesh of 15 × 15 × 1 *k* points was used for relaxation of one unit cell, while a 50 × 50 × 1 mesh was used for calculation of the momentum matrix elements in *k*-space. A vacuum of ≈11 Å was added to avoid interactions between periodic images of the system.

**Fig. 1:**
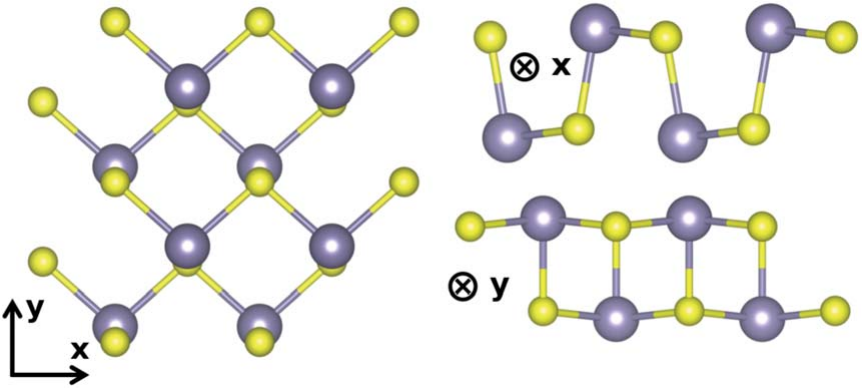
Armchair and zigzag directions adopted in this work.

The lattice parameters found with these settings are shown in Table 1. We follow the same conventions with *x* the zigzag and *y* the armchair direction (Fig. 1) as reported in Lin *et al.*^10^ In SnS, the extra valence electrons of S are pushing the three bonds of the atom towards a tetrahedral coordination.^23^ The SL pseudopotential resulted in values close to previously reported for DFT-GGA,^17,23^ while the PAW pseudopotential, that included the core Sn 4d states, resulted in the longest distance between the unbonded Sn–S in the chain, as well as larger lattice constant (*a*).

**Table 1:** Electronic bandgap energies (in eV) at the different points in the BZ and lattice parameters for each pseudopotential used. For the bandgap values, Indirect (I) is for *ΓY* valence to the *ΓX* conduction valley and Direct (D) is for the *ΓY* valleys

Pseudo	Bandgap	*ΓX*	*ΓY*	*Γ*	*a* (Å)	*b* (Å)
PAW	1.50 (D)	1.71	1.50	1.85	4.12	4.25
SL	1.39 (I)	1.66	1.51	1.81	4.08	4.21

Detailed bandgap values are also shown in Table 1. These were again consistent with previous work with GGA for the SL pseudopotential,^23^ which was indirect from the *ΓY* valence to the *ΓX* conduction valley.^17,23^ The PAW pseudopotential resulted in slighter larger bandgap as well as a transition to a direct bandgap, with only 20 meV difference, however, between the bottom of the conduction band in the *x* and *y* axes. Due to the small differences between the bandgap energies in different sections of the Brillouin Zone (BZ), most authors mention all bandgap energies^5,8^ as small changes in the chosen methods in DFT can lead to changes in the position of the smallest bandgap. This further aids in the analysis of optical bandgaps with the use of selection rules.

Overall, both pseudopotentials overestimated the lattice constants, which is expected for DFT-GGA.^24^ The PAW pseudopotential resulted in larger values of the electronic bandgap which could be attributed to the inclusion of the 4d core states in the valence. However, it was also observed that after Wannierization, the symmetry of the orbitals was not preserved when comparing the orbital projected band structure at the edges of the BZ (see ESI†). Therefore, further in this work, the PAW pseudopotential was used for deriving the momentum matrix elements in DFT, while the SL pseudopotential was used for the Wannierization procedure.

**Fig. 2:**
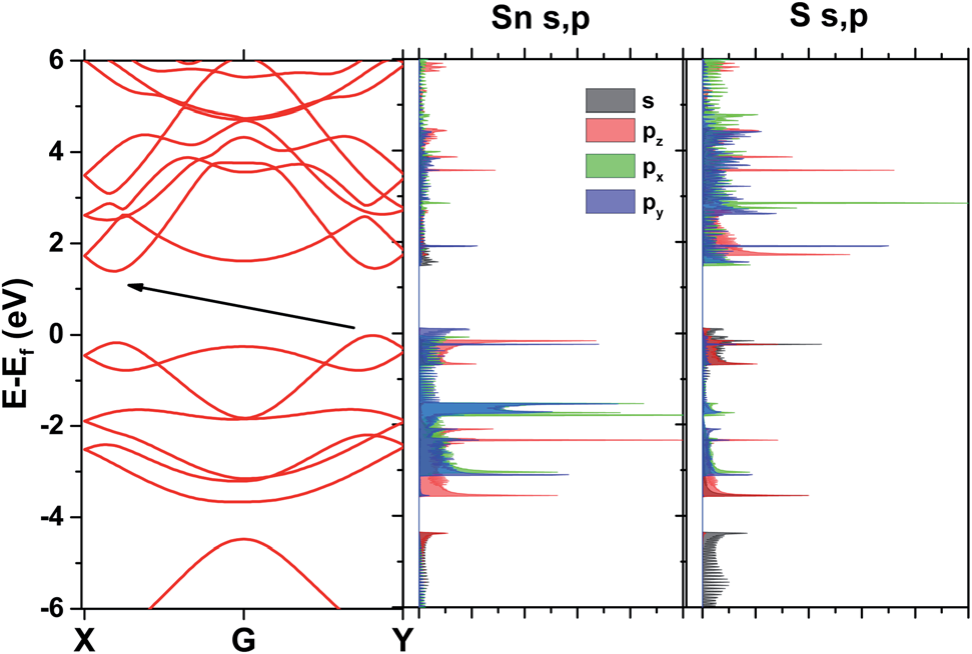
Band structure and DoS from DFT with the SL pseudopotential.

Band structure results for the SL pseudopotential are shown in Fig. 2(a) and projected Density of States (DoS) in Fig. 2(b). The latter revealed that the conduction band minimum is primarily composed of the Sn p electrons and the maximum of the valence band of the S p electrons. However, Lowdin charges^25^ were also extracted to check the transfer of charge between the atoms and are shown in Table 2 for both pseudopotentials. In both cases, the charge seems to be moving to the p states of sulfur, reducing from the p states of Sn and the s states of both atoms.

**Table 2:** Lowdin charges for Sn and S atoms with PAW and SL pseudopotentials defined in relation to their initial configuration of electrons

		s	p	d
PAW	Sn	−0.1637	−0.5146	−5e–4 (4d)
PAW	S	−0.2103	+0.8301	—
SL	Sn	−0.295	−0.5861	—
SL	S	−0.2293	+0.7879	—

**Fig. 3:**
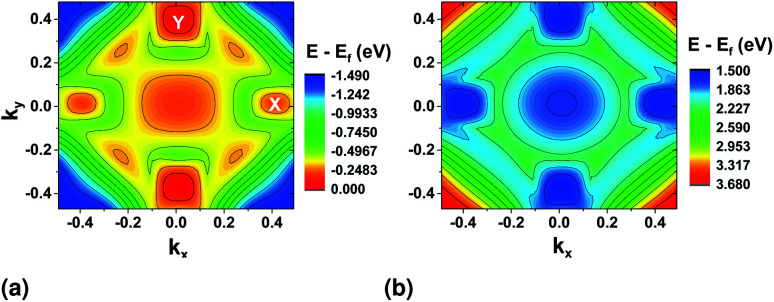
(a) Top valence band and (b) bottom conduction band surface plots from DFT with PAW pseudopotential.

Band profiles for the top most valence (VBM) and the bottom conduction (CBM) bands were also extracted from DFT and are shown in Fig. 3. The minimum/maximum locations show the positions where the electrons are most likely to make a transition from the valence to the conduction band combined with the selection rules set by the light polarization. The valence band shows clearly two maximum locations at the *ΓX* and *ΓY* sections of the BZ. In the valence band, the valley in the *x* direction is lower in energy than that in the *y* direction, while they are closer in energy in the conduction band.

## 3 Wannier functions

Wannier90 was used for the converting the Bloch wavefunctions to WFs.^26^ For both pseudopotentials, projected WFs and a minimization energy window that included the six top valence and six bottom conduction bands were used. The Bloch bands were projected onto the Sn and S atomic p states only. The spread functional for the gauge-invariant part was converged at a fractional change of 10^−9^ between successive iterations.

**Fig. 4:**
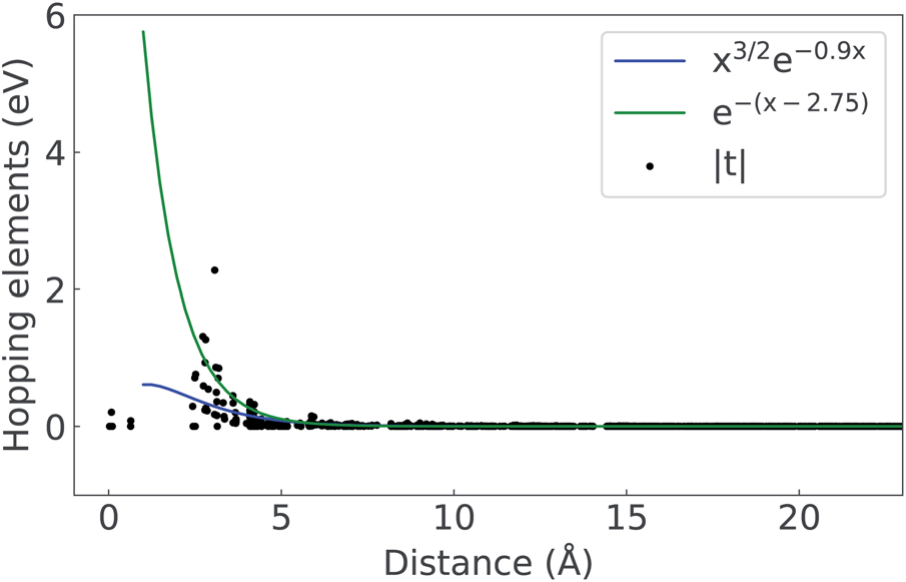
Absolute value of the hopping elements, |*t*|, as a function of distance from the Wannier center.

With these settings, a minimization of nearly zero Im/Re ratios was achieved, while proper representation of the Bloch states was also checked from the band structure results. The localization achieved for the Wannier functions has eigenenergies and bandgap dependencies,^27^ with the known problem of non-existence of maximally localized Wannier functions for topological materials.^28^ To check the localization of our projected WFs, the magnitude of the hopping elements as a function of distance from the WF center was plotted in Fig. 4 (ref. 29) and compared against two analytical values (i) *x*^*a*^e^−*hx*^ with *a* = 3/2 and *h* = 0.9 (ref. 27) and (ii) e^−(*x*−*d*)^ where *d* = 2.75 is the average bond length in the structure.^30–32^

**Fig. 5:**
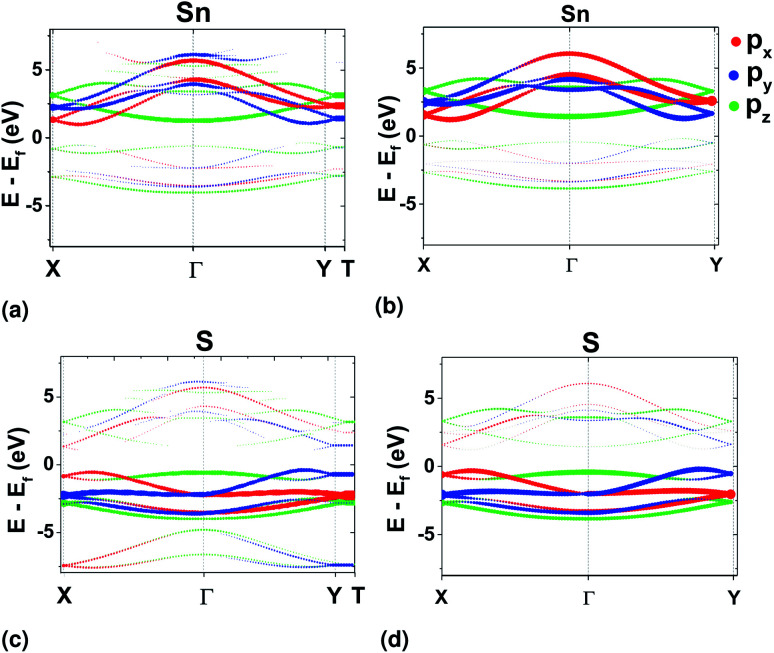
Orbital projected band structure results for Sn from DFT (a) and Wannier (b). Equivalently, for S from DFT (c) and Wannier (d). The pseudopotential used in the DFT calculations was the SL GGA.

For a representative tight-binding model in the Wannier basis, preservation of the character of the bands after Wannierization has to be assured. This was checked using orbital projected band structure calculations at the edges of the BZ and comparing with those in DFT (Fig. 5). It was found that only with the SL pseudopotential the character of the bands was correctly preserved in *ΓX* and *ΓY* directions, while with the use of the PAW pseudopotential, the valleys acquired p_*x*_ and p_*y*_ character of equal magnitude in both directions (see ESI†). It should, however, be noted that there are other means of preserving symmetries with the WFs, which were not attempted in this work.^33,34^

## 4 Optical transition probabilities

For the possibility of an interband transition to occur in a specific section of the BZ due to the interaction with the electromagnetic field of the wave, combined with the band structure surface results, we further need to look at the matrix elements of the momentum operator, p̂_*x*,*y*_, for that specific direction from the DFT results, equivalent to the electric dipole moment for the transition,

2 |〈*ψ*_i_|p̂_*x*,*y*_|*ψ*_f_〉|^2^

where the subscript i and f are for the initial and final states from the top of the valence band to the bottom of the conduction band.^8,11^ In the Wannier representation, these states are replaced by the WFs, *u*_i,f_, and we take the velocity matrix elements,^35^3
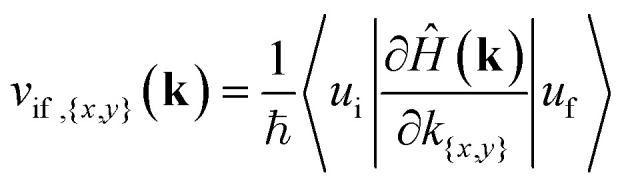


**Fig. 6:**
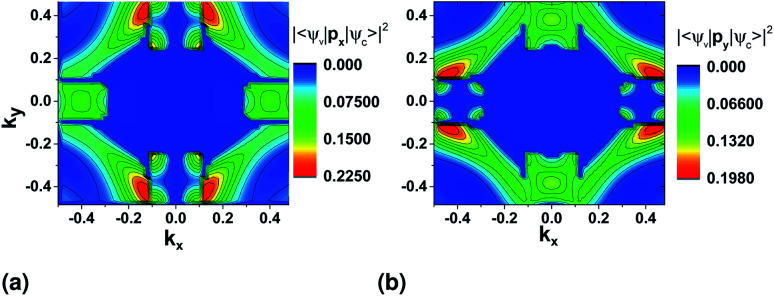
Momentum operator matrix elements for the VBM to CBM transition with direction (a) p̂_*x*_ and (b) p̂_*y*_ derived using DFT and the PAW pseudopotential. **p**_***x***_ and **p**_***y***_ is the momentum operator in the *x* and *y* direction respectively.

We first look at the momentum matrix elements from the VBM to CBM derived from DFT (Fig. 6). They are almost identical images of each other rotated by 90°. The areas of most interest are the two valleys in the *ΓX* and *ΓY* where the probability amplitudes are also higher (*ΓX* for p̂_*x*_ and *ΓY* for p̂_*y*_), confirming the expectation for optical transitions in the two direct bandgaps of the *x* and *y* axes (also see band structure surface plots in Fig. 3).

There is also two areas where the expectation value is higher around the valleys on each transverse axis. However, these correspond to allowed indirect transitions, that have much lower occurrence rate. The involvement of phonons for these cases further lowers the rate in SnS monolayers, but could be more important in the case of bulk SnS.

For both pseudopotentials, the expectation value of p̂_*x*_ is nearly zero in the *y* direction, but not of p̂_*y*_ in the *x* direction. The latter shows a small, finite value which results in an overall polarization ratio (*ΓY*/*ΓX* valley) of 25.5 for *y*-polarized light. This can manifest as reduced intervalley polarization degree for the *ΓY* valley in photoluminescence experiments^10^ of monolayer SnS.

**Fig. 7:**
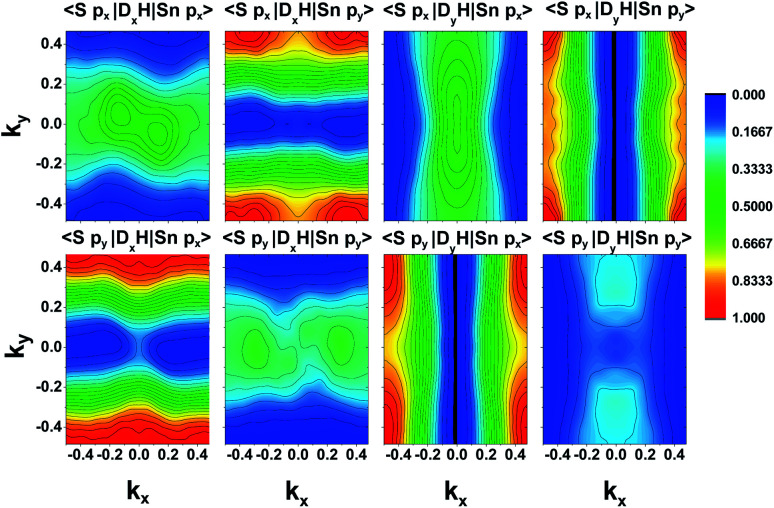
Velocity matrix elements for the Wannier functions of the Sn and S p_*x*_ and p_*y*_ orbitals.

To have a more detailed image of the contribution of each orbital to the magnitude of the momentum matrix elements, we modified the Wannier90 code to visualize the velocity matrix elements for each orbital (eqn (3)), as shown in Fig. 7, where *D*_*x*,*y*_*Ĥ* is for 
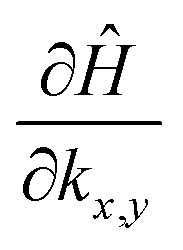
. While the major contribution for each direction of the velocity operator comes from the equivalent orbitals (p_*x*_ for *ΓX* and p_*y*_ for *ΓY*) between S in the valence and Sn in the conduction band, however, we can see how 〈Sp_*x*_|*D*_*y*_*Ĥ*|Snp_*x*_〉 and 〈Sp_*y*_|*D*_*x*_*Ĥ*|Snp_*y*_〉 are higher in the opposite directions for *k*_*x*_ = 0 and *k*_*y*_ = 0 respectively and at the edges of the *k*-space for transitions between orbitals with orthogonal orientation. The latter could explain the high values of the momentum matrix elements derived from DFT at the regions of indirect bandgap transitions (Fig. 6), provided that these orbitals have indeed contribution to the CBM and VBM bands at the specific locations of the reciprocal space.

Although the final result is primarily dictated by the projection of each orbital in the band structure (Fig. 5), the velocity elements from Wannier functions can also reveal information about the origin of intersubband transitions, which was beyond the scope of this work.

## 5 Ferroelectricity and valley selection

SnS exhibits spontaneous polarization as well as ferroelectric properties.^36^ When an electric field on the order of ≈0.1–0.3 V nm^−1^ is applied in the armchair or zigzag directions of monolayer SnS, the puckering direction rotates by π/2^8^. In the same work, the authors used this feature, as well as optical selection rules to detect the orientation of the structure.

**Fig. 8:**
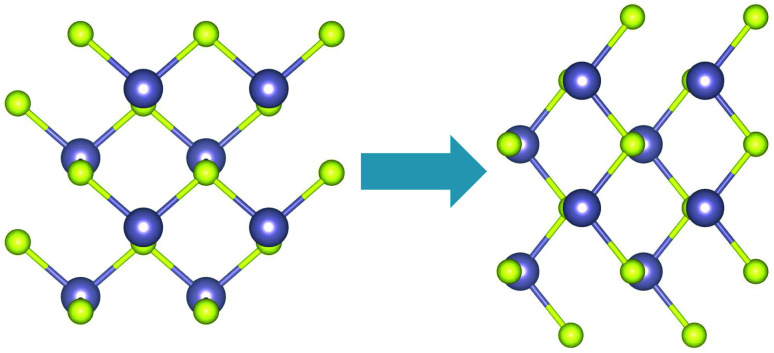
Rotation of the sulfur atoms under application of electric stress in the *x* direction (right).

In this work, the PAW pseudopotential was used for testing rotation of the atoms with the application of the electric field in the zigzag direction. The electric field was simulated based on the Quantum Espresso implementation of the methods described in^37,38^ and was applied during relaxation where only the atoms were allowed to relax to their final positions. The tolerance for total energy minimization was reduced to 10^−5^ eV. After progressively increasing the magnitude of the electric field with a step of 0.1 V nm^−1^, we found a critical field of 0.3 V nm^−1^ for rotating the symmetry of the system (Fig. 8).

**Fig. 9:**
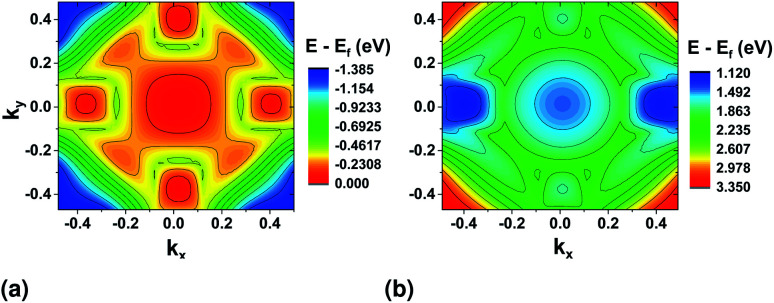
Valence band (a) and conduction band (b) surface plots after application of electric stress in the zigzag direction. DFT calculations were performed using the PAW pseudopotential.

The magnitude of the field chosen for further simulations is higher than this critical field (0.718 V nm^−1^). The resulting band structure surface plots are shown in Fig. 9. For the top valence band (Fig. 9(a)), when compared to the band structure without stress (Fig. 3(a)), the valley at *ΓX* moves higher in energy. The highest point remains at *ΓY* (≈32 meV difference). For the bottom conduction band, the valleys in the *y* direction nearly vanish, and the bandgap becomes indirect from *ΓY* to *ΓX*.

**Fig. 10:**
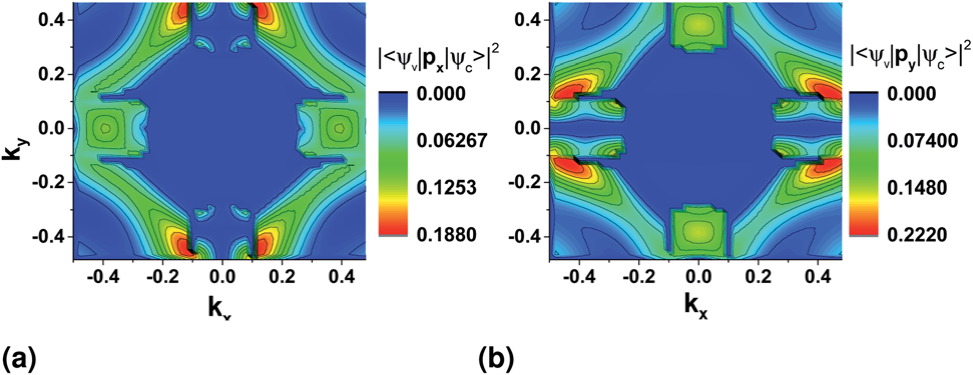
Momentum operator matrix elements for the VBM to CBM transition with direction (a) p̂_*x*_ and (b) p̂_*y*_ derived using DFT results and applying electric stress of 0.718 V nm^−1^ in the zigzag direction. The PAW pseudopotential was used in this case.

The valley polarization was checked by plotting the momentum matrix elements from DFT, as previously (Fig. 10), where there is an obvious increase in the magnitude of the expectation value of p̂_*y*_ along the *k*_*y*_ = 0 and a decrease for p̂_*x*_ along *k*_*x*_ = 0. For the direct bandgap transitions, however, the expectation value follows the opposite trend: a small expectation value of p̂_*x*_ appears at the *ΓY* valley which is equivalent to the non-zero expectation value for p̂_*y*_ at the *ΓX* valley before application of the electric stress.

The ratio of polarization with *x*-polarized light for the *ΓX* over that in *ΓY* valley after the application of the electric field is ≈40.3, while polarization of the *ΓY* valley with *x*-polarized light becomes impossible (zero matrix element). This behaviour is similar to that observed by Hanakata *et al.*^8^

The change in the polarization efficiency of the valleys with the application of electric stress would need to be considered in device configurations with electrically-induced valley selection. It should also be noted that previous work has estimated that rotation can happen up to a 5-monolayer structure, beyond which the barrier becomes too high for the atoms to rotate.^24^ Similar work on nanoribbons is however still missing in the literature. At the same time, as mentioned in the introduction, valley polarization has been reported for both bulk^10^ and 50 nm thickness SnS flakes,^11^ whose valley configurations are not affected by application of electric stress.

## 6 Tight-binding model in nanoribbon configuration

When using electrical means to probe valley separation, we are interested in finite size of SnS 2D structures. This stems from the fact that the transverse current at the *ΓX* valley will be cancelled by that of the *ΓY* valley, unless it is carried at the sides of the sample,^9^ where only one the of valleys contributes to the current. Therefore, we next consider a nanoribbon configuration of SnS.

Finite-size nanostructures present distinct properties from their 2D counterparts. These can be due to charge reconstructions^39^ or from pure geometrical effects.^40^ In SnS, it has been shown that the bandgap can be tuned by changing the thickness of the material,^41^ while in a bare nanoribbon configuration, metallic edge states appear in the zigzag direction.^42^ Here we show that the optical bandgaps can be tuned by changing the width of the nanoribbon using an effective tight binding model derived from the Wannier representation.

The band structures resulting from nanoribbons of 1–10 unit cells (40–42 Å) wide were calculated using the Tight Binding (TB) model derived in the WF basis. TB models exclude any charge reconstruction and related effects but give a good sense of the role geometry plays, in this case for the existence of the valleys in the band structure.

**Fig. 11:**
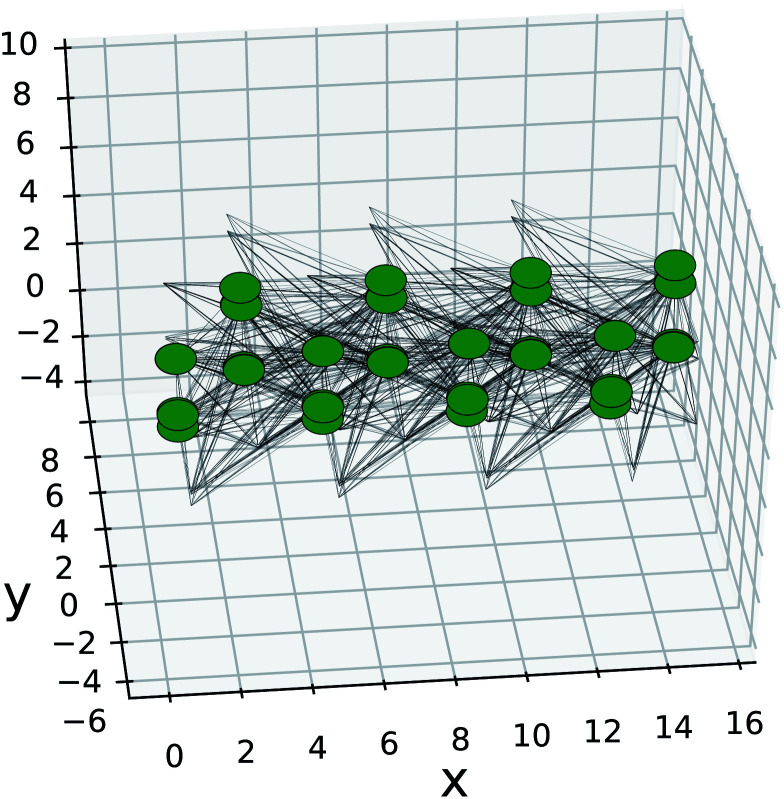
Kwant SnS model of nanoribbon with translational symmetry in the armchair direction and width of 4 unit cells. The green dots are the Wannier centers for each orbital, black lines are the hopping elements. The edges that lack nodes are for the next-nearest neighbour connections to orbitals that are repeated infinitely in the armchair direction.

**Fig. 12:**
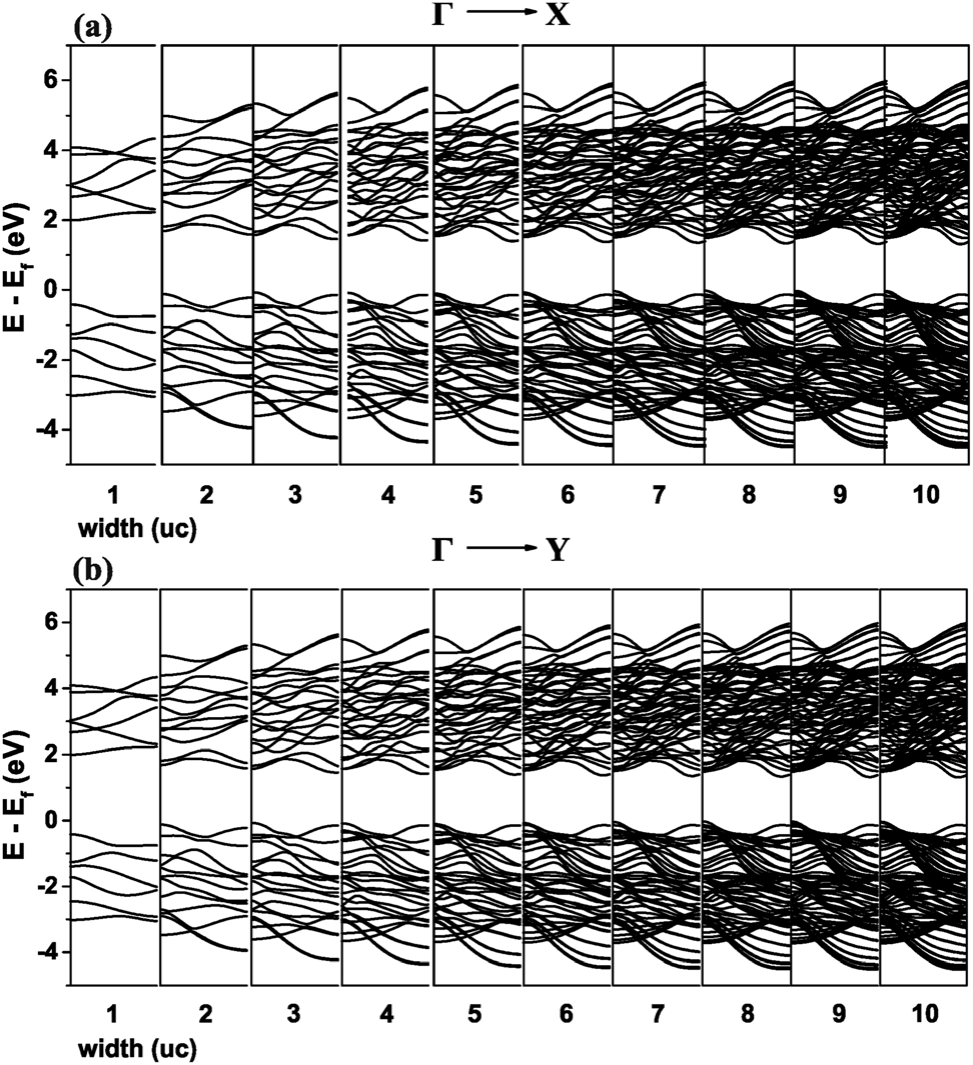
Band structure of the nanoribbon from the TB model for the (a) *ΓX* and (b) *ΓY* directions of the BZ. The width of the nanoribbon denoted below each graph is in number of unit cells.

The Kwant software package^43^ was used for creating the nanoribbons and the on-site energies and hopping elements were extracted from Wannier90 using TBmodels.^28^ Only next-nearest neighbours were preserved as the hopping elements were already found to be negligible beyond that (Fig. 4). The structure created in Kwant is shown in Fig. 11. The nanoribbon is constructed by imposing periodic boundary conditions for each direction separately and choosing the appropriate width. The resulting band structures are shown in Fig. 12.

Significant changes in the band structure as the width of the nanoribbon reduced below 5 units cells are observed. It is evident that the two valleys on the *x* and *y* axes are gradually reduced and eliminated for the case of one unit cell.

**Fig. 13:**
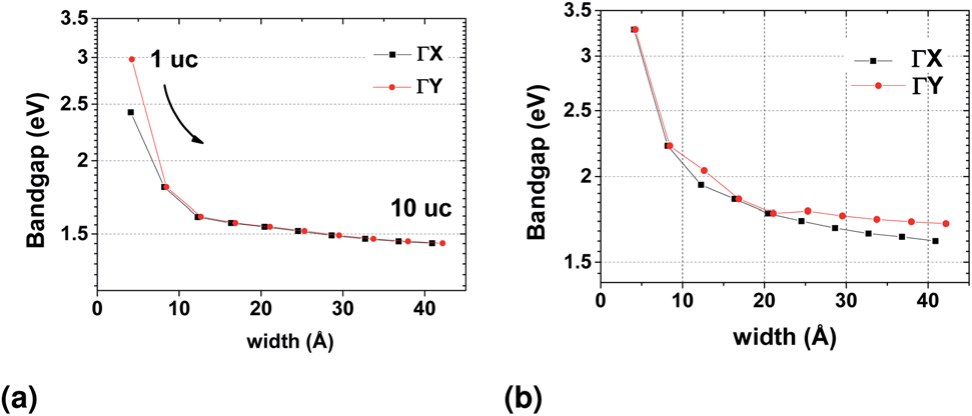
Evolution of the bandgap with nanoribbon width from the TB model in the Wannier basis where the Bloch wavefunctions are projected on (a) p orbitals only (b) both s and p orbitals of the atoms.

Because of the small but non-negligible contribution of the s orbitals of Sn (Fig. 2), we also constructed WFs that included all s orbitals in the basis (see ESI†). Both results are shown in Fig. 13. The two TB models are qualitatively similar, with only the magnitude of the bandgap being closer to that calculated from DFT results when the s orbitals are included. The bandgap in both cases converges to a stable value as the width of the nanoribbon increases.

As already mentioned, the width of the nanoribbon should be such that it will allow valley current of opposite direction to accumulate at the sides of the sample, as in the bulk, the contribution from each valley will cancel the other. From this point of view, the geometry of the sample does not seem to affect the characteristics of the final device, as long as its width is kept such that the bandgap values at the *x* and *y* directions are restored to those of the infinite 2D structure. For the cases where we are optically probing valley polarization, our results would be translated to decreasing absorption spectra for both directions of linearly polarized light when the nanoribbon approaches a single unit cell in width.

We generally observe that it is possible to tailor the optoelectronic properties of the nanoribbon by controlling its width. When compared to equivalent DFT results,^42^ the absence of metallic edge states is a further proof that the latter are a result of charge reconstruction at the edges, and unrelated to geometry effects of the structure.

## 7 Conclusions

Emergence of valley selectivity for optical transitions has been described with the use of the momentum operator matrix elements in *k*-space from DFT results in monolayer SnS. Use of Wannier functions was also made for deriving an effective TB model for the description of the system. A Troullier–Martins semi-local pseudopotential was found most appropriate for this purpose, as it preserved the characters of the bands at the top valence and bottom conductions bands.

Electric stress was also applied in the zigzag direction of the structure, which resulted in rotation of the atoms and a nearly vanishing *ΓX* valley in the conduction band. This theoretical treatment revealed a nearly perfect polarization degree for the *ΓX* valley with *x*-polarized light before the application of electric stress, and for the *ΓY* valley with *y*-polarized light after the application of electric stress.

The TB model was then used for calculating the band structure of SnS nanoribbons up to 40 and 42 Å. The model was qualitatively correct when only the p orbitals of the atoms were included, but only with the inclusion of the s orbitals it was also quantitatively equivalent to the DFT results. The valleys are lost when 1D translation symmetry was imposed on the structure that resulted from the TB model when just one unit cell was included, but gradually restored as the width of the nanoribbon increased.

This work can be used to further create computational models for calculating the transverse current in valley filter devices using quantum transport methods from first principles. It also revealed the possibility of fine-tuning the bandgap of SnS for optoelectronic applications by controlling the width of the nanoribbons constructed.

## Conflicts of interest

There are no conflicts to declare.

## Supplementary Material

NA-001-C9NA00555B-s001

NA-001-C9NA00555B-s002

NA-001-C9NA00555B-s003

NA-001-C9NA00555B-s004

NA-001-C9NA00555B-s005

NA-001-C9NA00555B-s006
