# Supplementary material for: Emergence of valley selectivity in monolayer tin(ii) sulphide
Source: Nanoscale Adv. 2019 Nov 11;1(12):4863–9. doi: 10.1039/c9na00555b (PMC9417898; doi:10.1039/c9na00555b)
Supplement: NA-001-C9NA00555B-s004 [file NA-001-C9NA00555B-s004.pdf]

Supplementary information for 'Emergence of valley selectivity in monolayer tin(II) sulphide'

E. Chatzikyriakou* and J. Kioseoglou*

Department of Physics, Aristotle University of Thessaloniki, 54124 Thessaloniki, Greece.

E-mail: elchatz@auth.gr; sifisl@auth.gr

Wannier

The projected wannier orbitals, as they were defined in the wannierization, for Sn and S are shown in Figure 1. The pseudopotential used in the DFT calculation in this case is the semi-local (SL), as mentioned in the main text. The orbitals have the correct shape and orientation, as expected.

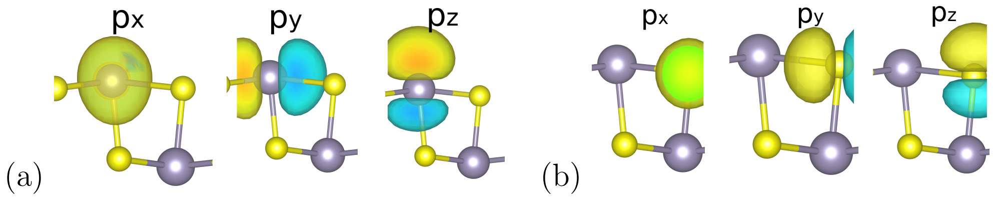

Figure 1: Wannier orbitals for (a) Sn and (b) S

Orbital projected band structure

Orbital projected band structures derived using the PAW pseudopotential are shown in Figure 2 for DFT and Wannier. After Wannierization, the p_x and p_y orbitals are overlapping, which is not the case in DFT. The symmetry of the structure is therefore lost. This requires re-examination of the wannierization procedure for derivation of a Tight-Binding model in a basis that preserves the character of the bands.

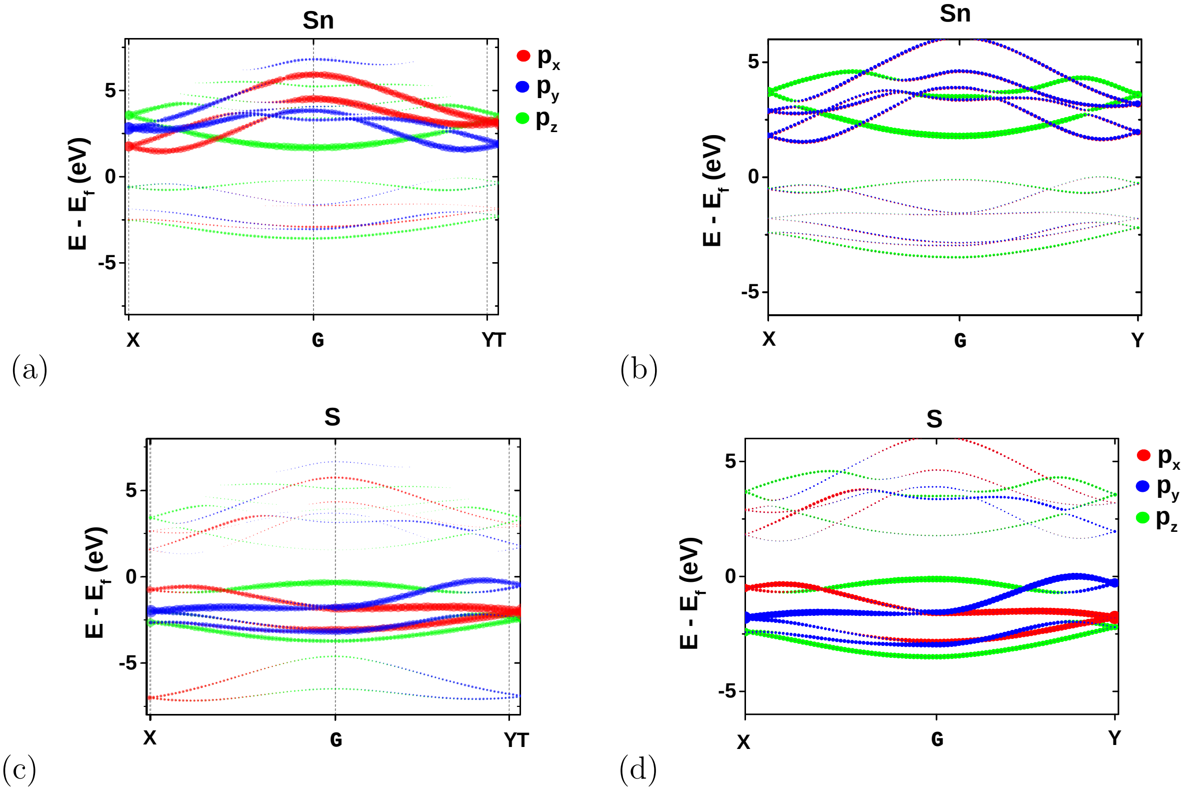

Figure 2: Orbital projected band structure results for Sn from DFT (a) and Wannier (b). Equivalently, for S from DFT (c) and Wannier (d). The pseudopotential used in the DFT calculations is PAW.

Velocity matrix elements in Wannier representation

The Wannier functions (WFs) that included the Sn s orbitals were derived by repeating the same procedure for the projected WFs from the DFT results with the SL pseudopotential as described in the main text, where the minimization window now includes the s states of both S and Sn. The projected band structure of the Sn s states from DFT and WFs is shown in Figure 3 confirming that the s states are well-represented in the Wannier basis in terms of the characters of the bands. The plots reveal that the s states also contribute to the two valley in the x and y directions, although with a lower density.

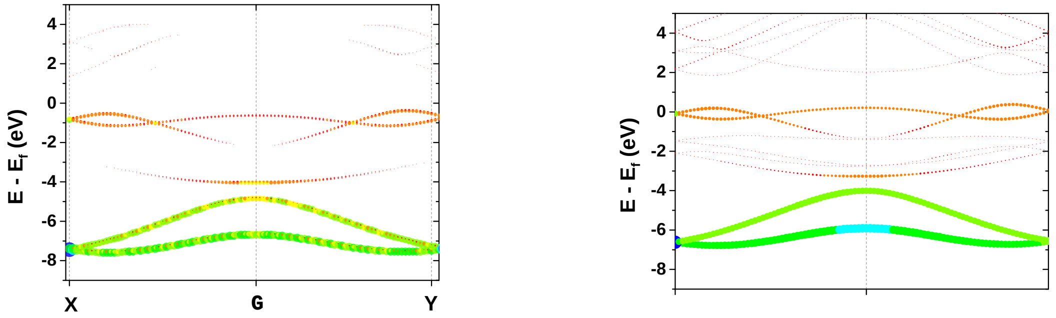

(a) (b)

Figure 3: Orbital projected band structure at the edges of the Brillouin zone for the s orbitals of Sn derived from (a) DFT and (b) Wannier results, with the SL pseudopotential in DFT.

Figure 4 shows the velocity matrix elements in the Wannier basis, for transitions between s, p_x and p_y orbitals of Sn and p_x and p_y of sulphur. Transitions $\left\langle Sn, s|D_x\hat{H}|Sn, p_x\right\rangle$ and $\left\langle Sn, s|D_y\hat{H}|Sn, p_y\right\rangle$ further confirm the shape of the relevant momentum matrix elements derived from DFT in the unstrained case in the main text. Namely, they show a non-negligible expectation value related to the direct optical transitions at the ΓX and ΓY valleys. The plots of the p_x and p_y orbitals remain the same as in the case where the s states are excluded from the wannierization.

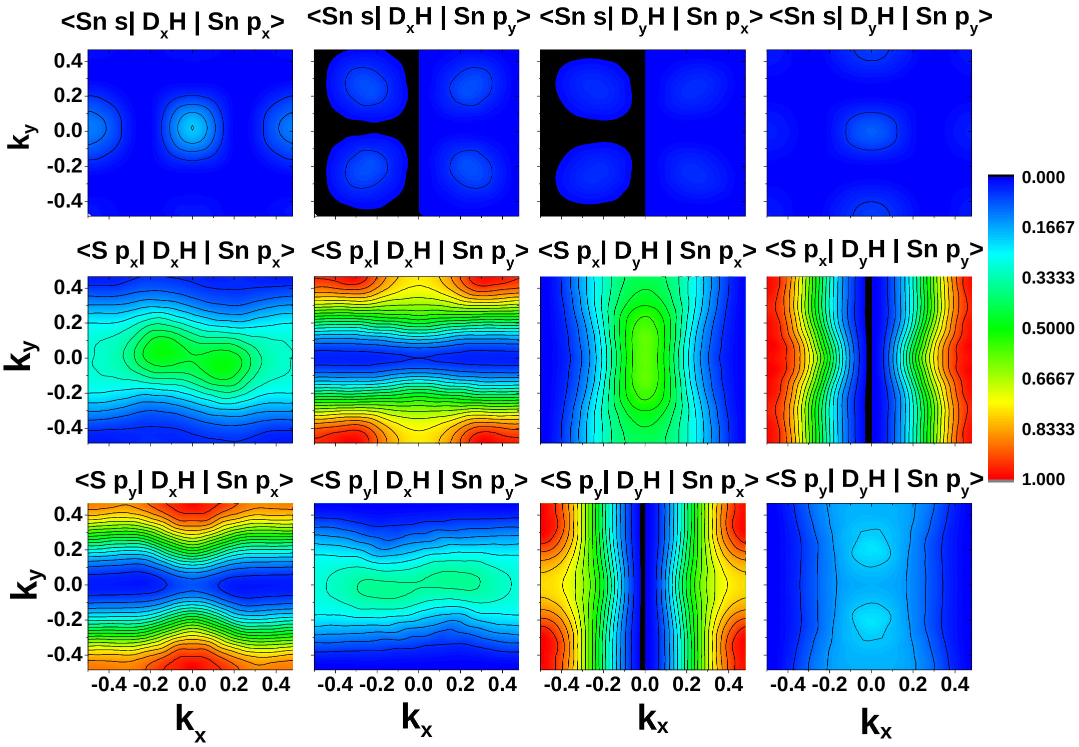

Figure 4: Velocity matrix elements for the wannier functions of the Sn s, p_x and p_y and S p_x and p_y orbitals. D_iH is for the partial derivative of the Hamiltonian $(\partial\hat{H}/\partial k_i)$ in the i direction.
